# TEMIS: A temperature-controlled microwave irradiation system for enhancing extracellular vesicle secretion

**DOI:** 10.1016/j.bbrep.2026.102608

**Published:** 2026-05-02

**Authors:** Haruki Odaka, Sho Nakazora, Hirofumi Soga, Hiroki Shimizu, Hiroaki Tateno

**Affiliations:** a Cellular and Molecular Biotechnology Research Institute, National Institute of Advanced Industrial Science and Technology (AIST), Tsukuba Central 6, 1-1-1 Higashi, Tsukuba, Ibaraki, 305-8566, Japan; b Technical Strategy Department, SHIKOKU INSTRUMENTATION CO., LTD, 200-1 Minamigamo, Tadotsu-cho, Nakatado-gun, Kagawa, 764-8502, Japan

**Keywords:** Microwave, Extracellular vesicles, Exosome

## Abstract

Extracellular vesicles (EVs) secreted by cells and tissues are promising tools for disease biomarkers and regenerative medicine, necessitating the establishment of technologies to efficiently enhance their secretion. Here, we investigated the effects of a temperature-controlled microwave irradiation system (TEMIS) on EV secretion from HEK293T cells. Unlike conventional microwave (MW) irradiation devices, TEMIS incorporates an active cooling mechanism that maintains the culture medium temperature at 37 °C during irradiation, thereby avoiding cell death associated with temperature elevation. Although cell viability remained unchanged, the amount of EV secretion in the MW-irradiated group increased by 2- to 3-fold compared to the non-irradiated control group, peaking approximately 3 h after irradiation began. No significant differences were observed between the irradiated and control groups in particle size distribution, proteome analysis, or miRNA expression analysis of the isolated EVs. These results indicate that MW irradiation can enhance EV secretion under temperature-controlled conditions, while preserving the molecular characteristics of the EVs. TEMIS enables MW irradiation without damaging cells, potentially making it a practical method for enhancing EV production from cells.

## Highlights

• TEMIS enables microwave irradiation while maintaining culture temperature at 37 °C.
• Temperature-controlled microwaves preserve HEK293T cell viability during exposure.
• Microwave irradiation increases extracellular vesicle secretion by 2- to 3-fold.
• Small EV yield increases without changes in size distribution or molecular profiles.
• TEMIS provides a non-destructive method to enhance EV production in cell culture.

## 1 Introduction

Extracellular vesicles (EVs) are nanoscale, membrane-bound particles released by nearly all cell types and are recognized as key mediators of intercellular communication [1]. Because EVs encapsulate proteins, nucleic acids, lipids, glycans, and other biomolecules reflective of their cellular origin, they have attracted considerable interest as platforms for biomarker discovery, drug delivery, and regenerative medicine [1]. However, a major limitation in their translational and industrial use is the intrinsically low yield of EVs obtained from cultured cells [2]. To overcome this challenge, numerous studies have explored methods to enhance EV secretion using various physical or chemical stimuli, including electric stimulation, irradiation with γ-rays, X-rays, ultraviolet, visible light, or sound waves, shear stress, heat stress, starvation, acidosis, heat shock, hypoxia, and oxidative stress [[2], [3], [4], [5], [6], [7], [8], [9], [10], [11], [12], [13]]. Although these approaches can increase EV secretion, they often do so by activating cellular stress responses, which can compromise cell viability and alter the molecular composition or biophysical properties of the produced EVs [2]. This presents a major challenge for large-scale EV manufacturing, where reproducibility, cell health, and product quality are critical.

Microwaves (MW) are a form of electromagnetic radiation with frequencies typically ranging from 300 MHz to 300 GHz [14]. Owing to their controllable energy delivery and ability to influence molecular processes, they are widely employed in industrial and scientific applications. In biological systems, MW exposure has been reported to modulate cellular phenotypes including alterations in cell growth, apoptosis, reactive oxygen species generation, DNA fragmentation, and modulation of intracellular signaling and metabolic pathways [[14], [15], [16]]. These observations raise the possibility that MW irradiation could serve as a non-contact physical stimulus for modulating cellular secretion processes. However, the potential application of MW irradiation in biotechnology and bioindustrial processes remains largely unexplored, particularly in the context of EV production.

In this regard, MW irradiation is of interest as a potentially controllable means of stimulating EV secretion. Nevertheless, a key challenge is that conventional MW exposure is often accompanied by temperature elevation, which can induce cellular damage and stress responses. For example, a recent study reported that brief exposure of tumor cells to short-wavelength MW heating enhanced the release of tumor-derived microparticles, but this effect was associated with apoptosis induced by thermal stress [17]. Thus, while this study highlights the capacity of MW-associated heating to trigger vesicle release, it does not address whether MW irradiation itself can enhance EV secretion under conditions that preserve cell viability. In other words, whether MW exposure can be harnessed as a practical and non-destructive strategy for EV production remains unknown.

To address this question, the present study investigates the effects of temperature-controlled MW irradiation on EV secretion, cell viability, and EV characteristics using a Temperature-controlled Microwave Irradiation System (TEMIS). By minimizing bulk heating through active cooling and evaluating both the quantity and molecular profiles of released EVs, we sought to determine whether MW irradiation can serve as a novel physical modality for enhancing EV production without compromising cell health or EV integrity.

## 2 Materials and methods

### 2.1 MW irradiation of HEK293T cells using the temperature-controlled MW irradiation system (TEMIS)

The experiments were conducted using HEK293T cells (CRL-3216, ATCC, Manassas, VA, USA), which are commonly employed for EV production. For the Tim4-*anti*-CD63 sandwich enzyme-linked immunosorbent assay (ELISA) experiments, cells were seeded in 6-well plates and cultured in Dulbecco's Modified Eagle's Medium (DMEM) supplemented with 10% fetal bovine serum until reaching a sub-confluent state. The medium was then replaced with 4 mL of DMEM (without NaHCO_3_, supplemented with HEPES). For EV isolation experiments, approximately 5 × 10^6^ HEK293T cells were suspended in 4 mL of DMEM (without NaHCO_3_, supplemented with HEPES) and transferred to 6-well plates. MW irradiation was applied using the temperature-controlled MW irradiation system (TEMIS), Aging Booster (Shikoku Instrumentation Co., Ltd., Kagawa, Japan) with a maximum output power of 50 W. The frequency was swept between 2410 and 2460 MHz in 256 steps, updated every 0.1 s in a bidirectional pattern, completing one cycle in 25.6 s. Output power was automatically adjusted to maintain the medium at 37 °C. At present, however, TEMIS does not directly measure electric field intensity or power density at the sample position. Because conventional SAR estimation requires a dielectric phantom and field mapping, these parameters could not be determined with the current setup. A temperature probe was inserted into the same culture well used for MW irradiation and EV collection to continuously monitor the medium temperature during exposure. To attenuate the rate of MW-induced temperature rise, a beaker containing 400–500 mL of distilled water was placed inside the irradiation chamber, serving as a thermal buffer. The chamber temperature was maintained at 4 °C, and the MW output was adjusted to keep the culture medium at 37 °C throughout the irradiation period. After irradiation, the culture supernatant was collected for subsequent downstream analyses. Cells incubated at 37 °C for the same duration without MW exposure served as the control group.

### 2.2 Tim4-*anti*-CD63 sandwich ELISA

The amount of EVs in the culture supernatant was quantified using a previously reported method with the PS Capture Exosome ELISA Kit (Streptavidin HRP; Wako Pure Chemical Industries, Ltd., Osaka, Japan), which employs a sandwich ELISA targeting two EV markers: phosphatidylserine and CD63 [18,19]. Because this assay detects phosphatidylserine- and CD63-positive vesicles, the measured particles are described here as EVs rather than exosomes, although they are enriched in small EV populations. Briefly, culture supernatants diluted 2- to 4-fold were incubated on Tim4-coated plates (a phosphatidylserine-binding protein) at room temperature (RT) for 2 h. After washing, biotinylated anti-CD63 antibody was added and incubated at RT for 1 h. Subsequently, horseradish peroxidase (HRP)-conjugated streptavidin was applied and incubated at RT for 2 h, followed by washing. Chromogenic reaction was carried out using 3,3′,5,5′-tetramethylbenzidine solution for 30 min, and the reaction was terminated by adding stop solution. Optical density (OD) was measured at a primary wavelength of 450 nm with a reference wavelength of 620 nm.

### 2.3 Isolation and nanoparticle tracking analysis of EVs

EVs were isolated from the culture supernatant using the MagCapture Exosome Isolation Kit PS (Wako Pure Chemical Industries, Ltd.), following the manufacturer's instructions. The size distribution and particle concentration of the isolated EVs were analyzed by nanoparticle tracking analysis (NTA). EV samples were diluted in phosphate-buffered saline and subjected to three independent measurements using the NanoSight LM10 system (Malvern Instruments Ltd., Worcestershire, UK).

### 2.4 Proteomic and miRNA profiling of isolated EVs

Proteomic profiling of EV samples was performed as follows. EVs were precipitated with acetone and dissolved in a protein solubilization buffer (8 M urea, 50 mM Tris-HCl, pH 8.0). The entire solution was subjected to tryptic digestion. Briefly, 2.2 μL of 100 mM dithiothreitol (DTT) was added (final concentration: 10 mM) and incubated at 37 °C for 30 min. Subsequently, 2.4 μL of 250 mM iodoacetamide (IAA) was added (final concentration: 25 mM) and incubated in the dark for 20 min. After reduction and alkylation, 55.4 μL of 50 mM Tris-HCl (pH 8.0) was added to reduce the urea concentration to 2 M. Finally, 1 μL of trypsin solution (0.5 μg/μL; final concentration: 12.2 ng/μL) was added, and the mixture was incubated at 37 °C for 16 h for enzymatic digestion. The resulting peptides were analyzed by LC-MS/MS for protein identification and quantification. MS/MS spectra were searched against sequence databases using the SEQUEST HT algorithm implemented in Proteome Discoverer (PD) 3.0 (Enzyme: Trypsin; Static Modification: Carbamidomethyl (C, +57.021); Dynamic Modification: Oxidation (M, +15.995)). Two databases were used: (1) UniProt entries for *Homo sapiens* (20,393 entries) and (2) a contaminant database from The Global Proteome Machine Organization. Peptide identifications were filtered using a false discovery rate (FDR) threshold of 0.01.

For miRNA profiling, total RNA was extracted from EVs using the 3D-Gene RNA extraction kit for liquid samples (Toray Industries, Kamakura, Japan) according to the manufacturer's instructions. RNA integrity was assessed using a Bioanalyzer (Agilent Technologies, CA, USA), and samples were labeled with the 3D-Gene miRNA labeling kit (Toray). Labeled RNA was hybridized to a 3D-Gene Human miRNA oligo chip (Toray), with probe annotations based on the miRBase database. After stringent washing, fluorescent signals were scanned using a 3D-Gene scanner and analyzed with 3D-Gene Extraction software. Raw signal intensities were normalized by subtracting the mean background signal (calculated from blank spots within the 95% confidence interval). Spots with signal intensities exceeding two standard deviations above background were considered valid. Relative expression levels of specific miRNAs were calculated by comparing normalized signal intensities across the array. Data were globally normalized so that the median signal intensity was adjusted to 25 for each array.

## 3 Results

### 3.1 TEMIS maintains normothermic conditions and preserves cell viability during prolonged MW irradiation

**Fig. 1:**
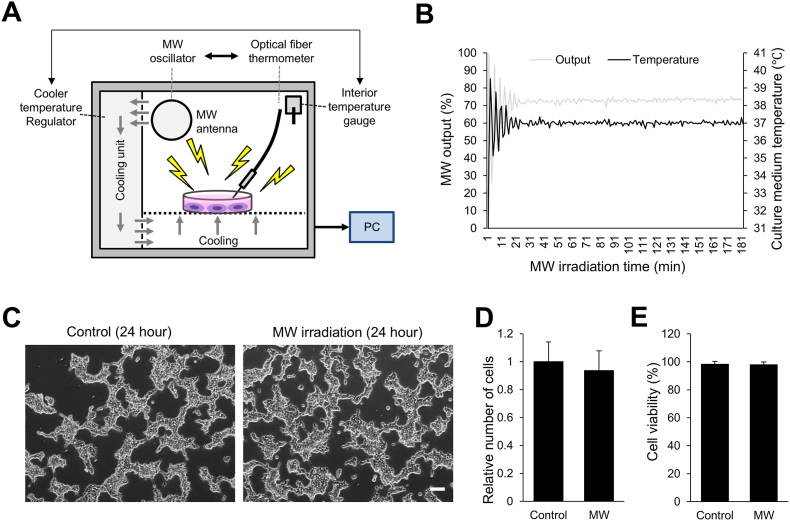
Effects of temperature-controlled MW irradiation on cell viability. (A) Illustration of the temperature-controlled MW irradiation system (TEMIS) for cell culture. (B) Time course of MW output and culture medium temperature during continuous MW irradiation. The medium temperature was maintained at 37 °C throughout the 3-h exposure period by actively adjusting the MW output. (C) Phase-contrast images of HEK293T cells under control conditions, and after 24 h of MW irradiation. Scale bar: 100 μm. (D, E) Quantification of relative cell number (D) and cell viability (E) after 24 h with or without MW irradiation, assessed using trypan blue exclusion. Control samples were measured in triplicate wells per experiment (a total of 9 data points), whereas one well per experiment was used for the MW-irradiated group (a total of 3 data points). Data represent mean ± SD of three independent biological experiments (n = 3). N.S., not significant (Student's t-test).

A schematic overview of the temperature-controlled microwave irradiation system (TEMIS) is shown in Fig. 1A. To determine whether TEMIS enables prolonged MW exposure under normothermic conditions, MW output and culture medium temperature were monitored during irradiation. As shown in Fig. 1B, the feedback-controlled system dynamically adjusted MW output while maintaining the culture medium at approximately 37 °C throughout exposure.

We next examined whether the temperature-controlled irradiation affects cell viability. HEK293T cells were cultured for 24 h under continuous MW irradiation. Phase-contrast imaging revealed that HEK293T cells exhibited a modest tendency toward aggregation as a result of the serum deprivation required for EV collection; however, no discernible differences in cellular morphology or confluency were detected between the control and MW-irradiated groups (Fig. 1C). To quantitatively assess cell viability, we measured total cell number and trypan-blue–based viability following MW exposure. Consistent with the morphological observations, no significant differences were detected between the MW-irradiated and control groups in either relative cell number (Fig. 1D) or cell viability (Fig. 1E). Together, these findings indicate that temperature-controlled MW irradiation does not adversely affect HEK293T cell growth or survival over 24 h under serum-free culture conditions, unlike conventional MW irradiation involving heating, which induces cell death [17]. These results confirm the successful establishment of a culture system that enables prolonged MW irradiation without adversely affecting cell viability.

### 3.2 MW irradiation enhances EV secretion and increases small EV yield without altering particle size distribution

**Fig. 2:**
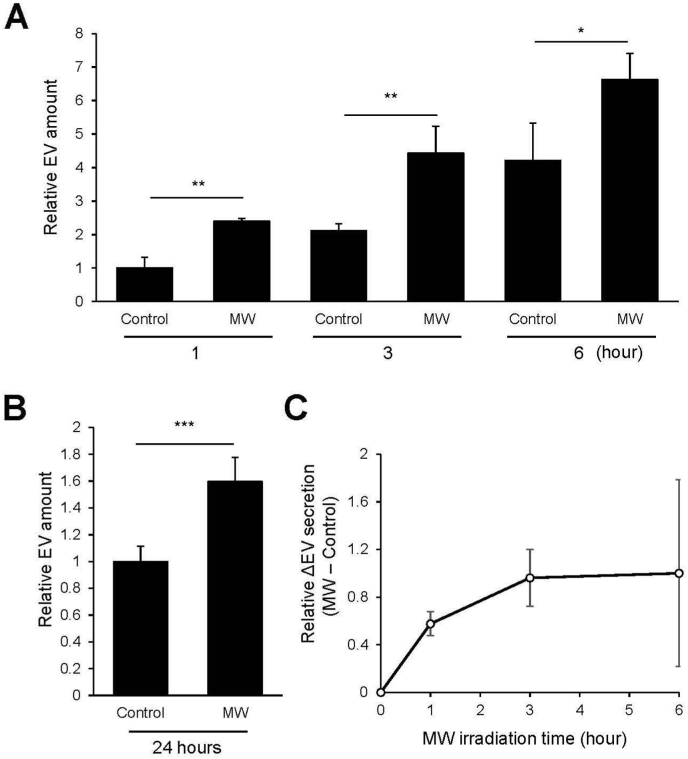
Effect of temperature-controlled MW irradiation on EV secretion. (A) Quantification of EV secretion in control and temperature-controlled MW-irradiated cells. Culture supernatants were harvested at 1, 3, and 6 h after the onset of irradiation, and EV abundance was measured using a Tim4-*anti*-CD63 sandwich ELISA. EV levels are presented as fold change relative to the 1-h control group (N = 3 per time point). (B) EV secretion at 24 h after MW irradiation, measured by Tim4-*anti*-CD63 sandwich ELISA and presented as fold change relative to the control group (Control: N = 7, MW: N = 3). (C) Time-dependent increase in MW-induced EV secretion. Data from panel (A) are expressed as the incremental EV amount (ΔEV = MW − control) at each time point, normalized to the ΔEV at 6 h (set to 1) (N = 3). Data represent mean ± SD. *p < 0.05; **p < 0.01; ***p < 0.001 (Student's t-test).

We next assessed whether MW irradiation affects EV secretion from cells. HEK293T cells were subjected to MW exposure, and EV abundance in the culture supernatant was quantified at 1-, 3-, and 6-h using the PS Capture Exosome ELISA. As shown in Fig. 2A, EV levels increased progressively over time in both groups; however, MW-irradiated cells consistently exhibited significantly higher EV secretion than non-irradiated controls at all time points, with increases ranging from approximately 1.6- to 2.4-fold (Fig. 2A). This increased EV secretion was sustained for at least 24 h following MW irradiation (Fig. 2B). To further evaluate the kinetics of MW-induced EV release, the incremental EV amount (ΔEV = MW − control) was calculated for each time point (Fig. 2C). This analysis revealed that the enhancement in EV secretion was most pronounced at 3 h after the onset of irradiation, after which the ΔEV plateaued. These findings show that MW irradiation substantially enhances EV secretion, with the strongest stimulatory effect occurring within the first several hours of exposure.

**Fig. 3:**
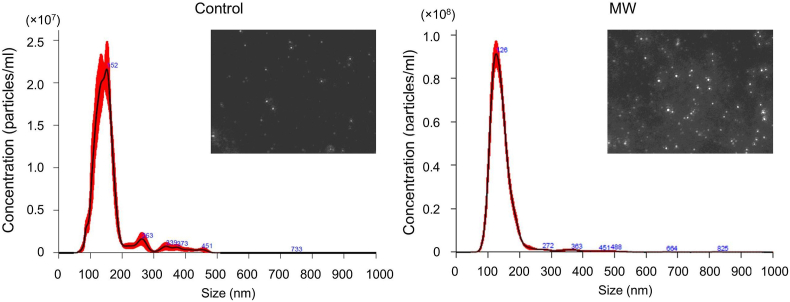
Size distribution and particle concentration of EVs following MW irradiation. EVs were isolated from culture supernatants harvested 3 h after MW irradiation using the MagCapture Exosome Isolation Kit. Particle size distribution and concentration were measured by nanoparticle tracking analysis (NanoSight). Representative NanoSight images are shown as insets within each panel for control and MW-treated samples.

To further determine whether the increased ELISA signal reflected an increase in secreted small EVs, EVs were isolated from culture supernatants collected after 3 h of continuous MW irradiation and analyzed by nanoparticle tracking analysis. The number of particles recovered from the MW-irradiated group was 5.95 × 10^8^ ± 2.01 × 10^7^, compared with 1.58 × 10^8^ ± 2.34 × 10^7^ in the control group, corresponding to a 3.77-fold increase in EV yield (Fig. 3). Despite the substantial increase in particle concentration, the size distribution profiles of EVs were comparable between the two groups. The mean particle diameters of the non-irradiated and MW-irradiated groups were 157.8 ± 4.5 nm (control) and 145.7 ± 1.3 nm (MW), respectively, indicating that MW irradiation did not appreciably alter EV size. Together, these results show that MW irradiation significantly enhances the yield of small EVs while maintaining their characteristic size distribution.

### 3.3 MW irradiation preserves the proteomic and miRNA profiles of secreted EVs

**Fig. 4:**
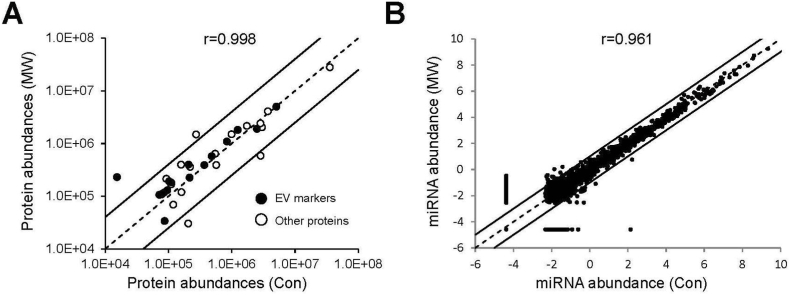
Effect of MW irradiation on EV protein and miRNA profiles. (A) Scatter plot comparing protein abundance in control and MW-treated EV samples. Each point represents an individual protein identified by LC-MS/MS analysis. Black circles indicate EV marker proteins listed in the ExoCarta database, whereas white circles denote all other proteins. The dashed line indicates the line of identity (y = x), whereas the two solid lines denote the ±4-fold change boundaries. Pearson's correlation coefficient (r = 0.998) is shown in the plot. (B) Scatter plot comparing miRNA abundance in control and MW-treated EV samples. Each point represents an individual miRNA species. The dashed line indicates the line of identity (y = x), whereas the two solid lines denote the ±2-fold change boundaries. Pearson's correlation coefficient (r = 0.961) is shown in the plot.

To evaluate whether MW irradiation affected the molecular composition of secreted EVs, we performed comprehensive proteomic and miRNA profiling analyses on isolated EV samples. LC-MS/MS-based proteomic analysis identified a total of 41 proteins after removing potential contaminants. Of these, 17 proteins corresponded to established small EV markers listed in the ExoCarta database, including tetraspanins (CD81), annexins (ANXA5, ANXA6), and heat shock proteins (HSP90AB1, HSPA8) [20] (Fig. 4A, Supplementary Table 1). The proteomic profiles of EVs from control and MW-treated cells showed a highly similar distribution of protein abundances, with protein expression levels exhibiting an exceptionally strong linear correlation between the two conditions (Pearson's r = 0.998, p < 0.001).

We next examined the miRNA cargo of EVs using a Human miRNA Oligo Chip. Among the 2632 detectable miRNA species, 270 exhibited more than a 2-fold difference between MW-treated and control groups (Fig. 4B–Supplementary Table 2). However, the majority of these miRNAs were of low abundance, and the overall miRNA expression landscape was strongly conserved between conditions, as reflected by a robust linear correlation (Pearson's r = 0.961, p < 0.001). Collectively, these findings suggest that EVs enhanced by MW irradiation possess proteomic and miRNA profiles comparable to those of control EVs.

## 4 Discussion

The present study shows that temperature-controlled MW irradiation substantially increases EV yield while preserving cell viability and the molecular integrity of secreted EVs. EVs collected from irradiated cells exhibited size distributions, canonical EV marker profiles, and miRNA signatures that were indistinguishable from those of control EVs, indicating that temperature-controlled MW exposure stimulates EV secretion, but does not perturb the fundamental physicochemical properties of secreted EVs. Importantly, continuous MW exposure under rigorously temperature-controlled conditions did not compromise cell viability, supporting the feasibility of applying this approach to prolonged or large-scale cultures. Together, these findings position temperature-controlled MW irradiation as a practical and scalable strategy to enhance EV production while maintaining the vesicle cargo profile, which is an essential requirement for diagnostic, therapeutic, and biomanufacturing applications.

A major limitation of this study is that the molecular mechanism underlying temperature-controlled MW-induced EV secretion remains unclear. Because the culture medium was maintained at 37 °C during irradiation, the influence of bulk heating and associated thermal stress can be minimized. In addition, no induction of heat stress–associated cellular responses, such as reduced viability or morphological alterations, was observed under these conditions. Previous studies have reported that non-thermal MW exposure can modulate intracellular Ca^2+^ signaling—a key regulator of EV biogenesis—suggesting a plausible link between electromagnetic field-induced calcium perturbations and enhanced EV release [21,22]. At the same time, localized heating cannot be ruled out, as our temperature monitoring system measures bulk medium temperature and does not directly detect microscale thermal gradients. In addition, MW interacts with polar molecules and ions, potentially generating microthermal gradients within cellular compartments [23]. Such localized temperature fluctuations may transiently alter membrane fluidity or activate stress-related signaling pathways, thereby facilitating vesicle budding and release [24,25]. Definitive separation of MW-induced and heat-induced EV release would require additional heat-only control experiments (e.g., incubator heating at 40–42 °C). However, it is well known that cell survival decreases markedly at such temperatures, making it difficult to apply thermal stimuli without simultaneously inducing significant cytotoxicity. While the present results indicate that classical bulk thermal effects are unlikely to account for the observed increase in EV secretion, the relative contributions of non-thermal electromagnetic effects and potential microthermal phenomena remain to be clarified in future studies. Although TEMIS minimized macroscopic heating by maintaining the medium at 37 °C, we were unable to experimentally verify the absence of localized microthermal effects with the current setup.

In addition, although the molecular composition of EVs was preserved, the biological functionality of MW-induced EVs was not assessed in the present study. In particular, whether these EVs exhibit equivalent therapeutic efficacy or biological activity compared with conventionally produced EVs remains unclear. It should also be noted that the present study was limited to HEK293T cells, which are widely used for EV production. Although current findings show the feasibility of TEMIS in this model system, its applicability to other cell types remains to be established. Future studies will be required to evaluate the functional properties of MW-induced EVs in relevant biological systems, and validation in diverse cellular systems will be necessary to assess the generalizability of this approach.

In this study, we developed TEMIS, a temperature-controlled MW exposure system without causing cellular damage. Furthermore, we found that TEMIS increases EV secretion without affecting their molecular characteristics. Future research is expected to elucidate the molecular mechanisms and utilize this system to promote EV production from various cells and tissues, thereby advancing EV research and industrial applications.

## Funding

This work was supported by SHIKOKU INSTRUMENTATION CO., LTD. under a research collaboration agreement, JST A-step (JPMJTR23U6), and JSPS KAKENHI (22H00585).

## CRediT authorship contribution statement

**Haruki Odaka:** Data curation, Formal analysis, Methodology, Writing – original draft. **Sho Nakazora:** Data curation, Formal analysis, Methodology, Writing – original draft. **Hirofumi Soga:** Conceptualization, Formal analysis, Methodology, Supervision. **Hiroki Shimizu:** Conceptualization, Data curation, Methodology, Supervision. **Hiroaki Tateno:** Conceptualization, Funding acquisition, Methodology, Project administration, Writing – original draft.

## Declaration of competing interest

The authors declare the following financial interests/personal relationships which may be considered as potential competing interests: Hiroaki Tateno reports financial support was provided by SHIKOKU INSTRUMENTATION CO., LTD. Hiroaki Tateno, Haruki Odaka, Sho Nakazora, Hirofumi Soga, and Hiroki Shimizu have a patent #PCT/JP2025/017755 pending to National Institute of Advanced Industrial Science and Technology, SHIKOKU INSTRUMENTATION CO., LTD. Hiroaki Tateno, Haruki Odaka, Sho Nakazora, Hirofumi Soga, and Hiroki Shimizu have a patent #PCT/JP2025/017752 pending to National Institute of Advanced Industrial Science and Technology, SHIKOKU INSTRUMENTATION CO., LTD. The authors declare that they have no other known competing financial interests or personal relationships that could have appeared to influence the work reported in this paper.

## Data Availability

Data will be made available on request.
